# Care planning for consumers on community treatment orders: an integrative literature review

**DOI:** 10.1186/s12888-016-1107-z

**Published:** 2016-11-10

**Authors:** Suzanne Dawson, Sharon Lawn, Alan Simpson, Eimear Muir-Cochrane

**Affiliations:** 1 School of Nursing & Midwifery, Flinders University, GPO Box 2100, Adelaide, 5001 Australia; 2 School of Medicine, Flinders University, Adelaide, Australia; 3 School of Health Sciences, Nursing, City University London, London, UK

**Keywords:** Community Mental Health, Community Treatment Orders, Case Management, Care Planning

## Abstract

**Background:**

Case management is the established model for care provision in mental health and is delivered within current care philosophies of person-centred and recovery-oriented care. The fact that people with a mental illness may be forced to receive care and treatment in the community poses challenges for clinicians aiming to engage in approaches that promote shared decision-making and self-determination. This review sought to gain an in-depth understanding of stakeholders’ perspectives and experiences of care planning for consumers’ on CTOs.

**Methods:**

An integrative review method allowed for inclusion of a broad range of studies from diverse empirical sources. Systematic searches were conducted across six databases. Following appraisal, findings from included papers were coded into groups and presented against a framework of case management.

**Results:**

Forty-eight papers were included in the review. Empirical studies came from seven countries, with the majority reporting on qualitative methods. Many similarities were reported across studies. Positive gains from CTOs were usually associated with the nature of support received, highlighting the importance of the therapeutic relationship in care planning. Key gaps in care planning included a lack of connection between CTO, treatment and consumer goals and lack of implementation of focussed interventions.

**Conclusions:**

Current case management processes could be better utilised for consumers on CTOs, with exploration of how this could be achieved warranted. Workers need to be sensitive to the ‘control and care’ dynamic in the care planning relationship, with person-centred approaches requiring core and advanced practitioner and communication skills, including empathy and trust.

**Electronic supplementary material:**

The online version of this article (doi:10.1186/s12888-016-1107-z) contains supplementary material, which is available to authorized users.

## Background

The concept that people should have a stronger voice in decisions about their health and care has been a policy goal in health for at least 20 years [1] with increased consumer involvement linked to improved care experiences and better clinical and economic outcomes [2]. In mental health care, case management is the established model for care provision and aims to integrate care and support across a broad range of services for individuals presenting with complex needs [3]. As there is no single definition of case management, for the purposes of this review, case management and care planning are explored utilising Ross et al.’s [4] framework of case-management with core components including: case-finding; assessment; care planning; care co-ordination and case closure.

Case finding in this review refers to consumers on a CTO. The care planning process, informed by ongoing assessment, should be personalised to the individual, address the range of issues that may impact upon their health and wellbeing and be co-produced with the person and relevant others involved in their care [4]. Care-coordination, ‘the essence of case management’ , requires case managers to collaboratively facilitate the above processes with the care plan the ‘live’ document recording this process [4]. Case managers working with consumers on CTOs have the additional role of managing the CTO requirements, which may include informing the consumer and family about CTO processes, participating in tribunal hearings, initiating recall to hospital and managing discharge from the CTO [5–7].

Central to case management in mental health is the therapeutic relationship, with positive associations found between ‘perceived patient involvement, satisfaction and empowerment’ [8, 9]. A recent systematic review examining barriers and facilitators to consumer involvement in care planning in mental health found consumer involvement was dependant on consumer capacity, the relational quality between consumers and health professionals and the organisational context, with the relational aspects of care planning most valued by consumers and their carers [8]. However despite benefits and policy support of increased consumer involvement, there has been limited progress towards fully involving people in their own health and care [1].

In mental health care a further challenge for clinicians is that forced care sits within service frameworks promoting recovery-oriented and person-centred care. The World Health Organisation state that ‘[p]ersons with mental health disorders should be provided with health care which is the least restrictive’ and that ‘maintaining legal instruments and infrastructures…to support community based mental health care’ is central to the implementation of this principle (p.8) [10]. Thus legal frameworks have been created to ensure individuals with a mental illness, whom are considered to pose a risk to themselves or others receive care and treatment through the use of CTOs [11]. Though CTOs typically last between 6 and 12 months, in reality many consumers will be on orders for extended periods [12] with rates of usage increasing in Australia [13].

Clearly challenges exist for mental health clinicians engaging consumers on such orders in ways that promote self-determination and empowerment. The issue of care planning with consumers on CTOs is complex, with CTO legislation, service delivery models and resource availability all impacting upon implementation [14]. Significant concerns regarding the effectiveness and ethics of CTOs also exist with a recent review examining CTO effectiveness finding no differences in social functioning, quality of life or service use for individuals on CTOs compared to those receiving standard voluntary care [15]. Advocates for CTOs cite clinical improvement and being the ‘least restrictive’ treatment option as benefits [16, 17], whilst advocates against CTOs, often ex- service users, consider forced treatment a major barrier to collaborative, person-centred care [18]. Further ethical concerns have been raised about current legislation for compulsory treatment in Australia where there is a lack of consideration of the individuals’ decision-making capacity [11].

In summary, though case management has been used in practice for several decades, there remains a lack of conceptual clarity of what personalised care planning is [19] and lack of evidence regarding its effectiveness [4, 20]. In mental health care, compulsory care further challenges concepts of personalised care planning. Over the past 20 years there has been significant debate in the literature about the purpose, value and stakeholder experience of CTOs. This review explores the impact of CTOs on case management. The intention is to add to the current evidence base with the aim of improving the process and experience of case management for all stakeholders, and specifically the experiences and outcomes for those consumers who find themselves on such orders. The integrative review method was the chosen methodology as it allowed for the inclusion of a broad range of studies from diverse empirical sources which was considered important in addressing this complex issue [21].

### Objectives

To gain an in-depth understanding of consumers’ , carers’ and mental health workers’ perspectives and experiences of care coordination and care planning for consumers on CTOs in community mental health settings.

## Method

### Search strategy

The search strategy, utilised for conducting Systematic Reviews, aimed to find published, peer reviewed literature relevant to the phenomena of interest [22]. An initial search with relevant keywords was conducted, followed by an extensive search from 2000 onwards with relevant keywords and index terms. Databases searched included: CINAHL; PubMed; Medline; Scopus; PsychINFO and ProQuest (see Additional file 1). Reference lists of papers meeting inclusion criteria were checked for additional papers and searches were registered with the databases, allowing for inclusion of papers published during data analysis. Studies of qualitative and quantitative design and opinion papers from any country were sought. Literature published from 2000 onwards was considered for inclusion to reflect current mental health care practice and mental health legislation pertaining to CTOs. Non-English papers and studies with forensic patient participants were excluded.

## Results

### Description of studies

**Fig. 1:**
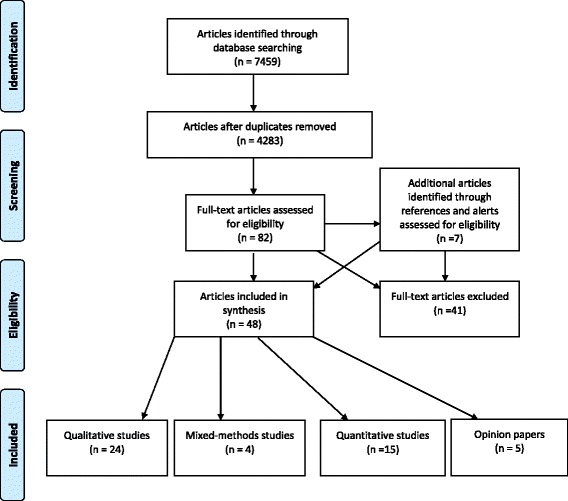
Flow chart of literature search strategy

A detailed search across selected databases identified 7459 papers. After removing duplicates, 4283 were examined against the objectives of the review and inclusion criteria by reading titles and abstracts. Eighty-two papers were retrieved for full review with a further 7 papers identified from reference lists and data base alerts. Forty-one papers did not meet the inclusion criteria and were excluded. A total of forty-eight papers were included in this review. Of the included papers, 24 reported on qualitative research, 15 on quantitative research, four used mixed-methods and five were opinion papers (see Fig. 1).

There has been a significant increase in publication of papers on this topic in recent years with 25 of the included papers published since 2013. Empirical studies came from seven countries: New Zealand [6]; UK & Scotland [9]; Australia [6]; Norway [3]; USA [4]; Canada [3] and Israel [1]. Often several papers reported on data from the same study. The highest number was seven papers reporting on a large qualitative study conducted in New Zealand. In this instance, although these papers all had a different focus, findings were considered conjointly when there was congruence across papers.

Studies reported on a variety of objectives though the majority explored the experience of CTOs from different stakeholder perspectives including consumers, carers and mental health professionals from varied backgrounds. Fewer studies included views of lawyers, advocates and members of mental health tribunals. More recently authors have reported on more nuanced issues related to care planning, though the majority of papers referenced the current policy environment of recovery-oriented care. Three studies aimed to interview key stakeholders involved in care planning. Gjesfjeld and Kennedy [23] interviewed consumers and their nominated mental health worker, and a large New Zealand study aimed to interview consumers, their case workers, psychiatrists and carers. Brophy and McDermott [24] explored the perspectives of people on CTOs, their carers’ , case managers and doctors, to inform best practice for individuals on CTOs. No study specifically explored the care planning relationship. With the exception of two studies that aimed to interview participants on two occasions [24, 25] to ascertain if participant views changed with time, all other studies collected data at one point in time.

### Quality of evidence

JBI appraisal tools relevant to study design were used, with key criteria selected from each of the tools [22]. Studies utilising mixed-methods were appraised against qualitative criteria as results relevant to the phenomena of interest were drawn from qualitative data. Overall qualitative studies were of good to excellent quality with good methodology and representation of participant voices. Common gaps were lack of stated philosophical perspective and lack of information about the researchers and their influence on the research.

Of the quantitative papers, only one paper reported on a randomised control trial [26]. This study has drawn much debate, though the authors’ clearly identify various limitations themselves, such as the inability for clinicians to persist with initial randomisation at subsequent stages of clinical decision-making. Also, given the participant group, it was not possible for participants to be blinded to treatment allocation or allocation concealed from the allocator. The remaining fourteen papers were descriptive or correlational case studies. In most studies the sampling would not be considered robust, with people volunteering to participate and no randomisation. Furthermore, measures used were not always validated, though this was considered appropriate given opinions were being sought.

All included opinion papers were written by individuals considered experts in the field of research regarding CTOs. No papers were excluded following appraisal.

### Availability of data and materials

The data supporting the conclusions of this article are included within the article (and in Additional file 2).

### Data synthesis

**Fig. 2:**
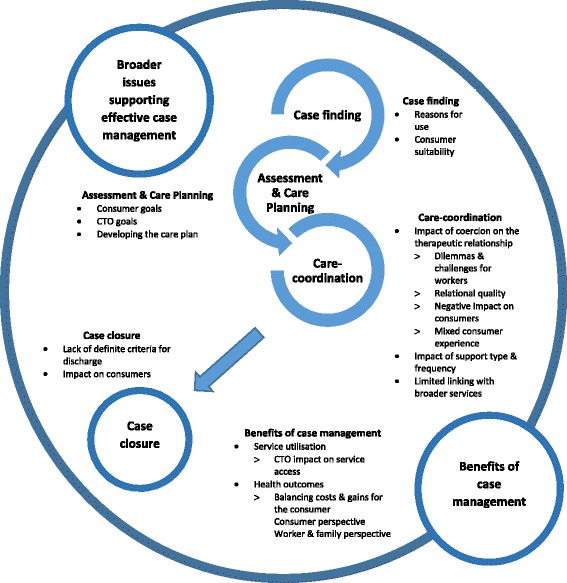
Framework for findings related to case management for consumers on CTOs

Data was extracted from the included papers and coded into categories using NVivo 10. These findings are presented against a framework of case management developed from Ross et al. [4] and includes: case-finding; assessment and care planning; care-co-ordination; case closure; benefits of case management and broader issues that support effective case management (see Fig. 2). The qualitative research and opinion papers, provided rich descriptive data, and form the main part of the synthesis, with data from the quantitative papers used to augment the findings.

## Core components of case management

### Case- finding

#### Reasons for use (positively and negatively framed)

This review includes individuals who are on a CTO and receiving case management from community mental health services. The reported reasons for consumers being placed on a CTO, from qualitative studies, included risk to self and/or others [27, 28], poor insight, lack of compliance with treatment (predominantly medication) [27, 29], ensuring treatment [28–30] facilitating discharge from inpatient services and hospital avoidance [29]. Quantitative studies reporting on workers perspectives of factors governing decision-making of CTO use found the most reported factors to be: ensuring contact with workers; protecting consumers’ from consequences of relapse; promoting medication adherence and providing authority to treat [31–34]. These clinical factors driving CTO use have remained consistent over the past decade, and across continents [31, 34].

#### Consumer suitability

Several studies highlighted the lack of usefulness of CTOs to those clients whereby coercion experienced from being on the CTO cancelled out any gains [35, 36] with clinicians reporting consumers most likely to benefit from a CTO being those with a level of insight into their mental health problems, and therefore more likely to collaborate with services [36, 37]. Consumers needed to accept the validity of a treatment order for CTOs to be a viable treatment option [37, 38] with Mullen et al. [39] suggesting that if good therapeutic relationships were not achieved within a reasonable period of time they should be discharged from the CTO to voluntary care.

### Assessment and care planning

Findings that relate to assessment and care planning are combined as in practice they co-occur, with ongoing assessment informing care planning. Key findings presented include consumer goals, CTO goals, development of the care plan and recommendations to improve assessment and care planning.

The care plan provides the framework for and documentation of the processes of assessment and care planning, though interestingly most studies did not directly reference the care plan. Of those that did, findings indicated consumers on CTOs had little knowledge about their care plan and what was in it with care plans often out of date and focussed on medication [25, 40, 41]. As the care plan is the means for recording the collaborative care planning process, Owens and Brophy [40] suggested that out dated care plans indicated that such conversations between clinicians and consumers may not be occurring regularly.

#### Consumer goals

Care plans should address consumer goals in the broader areas of social connection, community engagement and employment [5]. In practice however there was a lack of evidence of supporting consumers in these areas [42]. Instead, care plans were reported to be ‘formal records of deficits, professionally assessed needs, and allocated services’ (p.517) [25]. Reasons for CTOs were typically referred to as conditions, implying lack of consumer choice, and rarely linked to consumers’ recovery goals. In fact there was little reference to consumers’ goals, with consumers and carers expressing disappointment at the overly medical focus of the CTO and related care package [25, 29]. Only one study referenced links between CTO and consumer goals (in this case medication compliance linked with regaining a driver’s licence) [43]. Brophy et al. highlight the benefit of incorporating a more holistic perspective into the CTO process as ‘offer[ing] a valuable balance against the tendency to “over-medicalise” assessments of mental health problems and risk assessment’ (p.161) [44]. The same author highlights the diversity of consumers on CTOs as well as diversity in CTO goals or purposes and states that guidelines have tended to presume homogeneity amongst CTO recipients [24].

#### CTO goals

For the majority of participants in the included studies the primary purpose of the CTO was medication compliance, which then became the focus of interactions between workers and consumers [36, 45]. Workers and family members often linked the need for medication compliance with poor insight and increased risk of harm (to self or others) [46]. Interestingly, perceptions of risk differed amongst participant groups, with consumers and carers concerns focussed on the distress stemming from mental illness and subsequent social and interpersonal difficulties, and workers focussed on actual harm and potential risk [47]. Findings indicated that workers had variable thresholds for risk [39] with a worker in one study questioning what should be considered ‘normal’ , ‘at risk’ or ‘dangerous’ behaviour when informing the need for a CTO [45]. Other reasons or conditions for CTOs included the requirement to stay in specified accommodation [6, 43] and maintain contact with the mental health team [29, 48]. Consumer reports of CTO purpose varied with some unclear as to why they were on a CTO or what was being asked of them by services [23, 27], some perceiving they were on orders as a result of diagnosis or previous episodes, and reports of consumers and their mental health workers offering different requirements [23, 43, 49].

Whilst Banks et al. [48] warn that broadening CTO goals would be ‘ethically unsound’ , clarifying the purpose of the CTO and linking CTO goals with consumers’ individual recovery goals was considered necessary and achievable within care planning processes [5, 24]. Mental health workers’ stated CTO goals and conditions should complement care and treatment goals set out in the care plan [30], though workers also expressed scepticism and concern that CTOs could undermine the process of developing consumer led goals [50]. Given these challenges, Brophy and McDermott [24] recommend mental health professionals’ working with consumers on CTOs have advanced clinical and interpersonal skills.

#### Developing the care plan

When exploring findings to support collaborative care planning, it was found consumers often reported little or no opportunity to input into decisions related to the implementation of the CTO [25, 45, 48, 51, 52], though reported benefits (for example increased trust) from ‘being heard’ by clinicians, even when their views differed [48]. Increased input into decision-making was reported by some consumers at later stages of the CTO process, including reviews, as well as other care planning decisions (such as preferences for support options during crisis) [48, 53]. One study reported on the lack of impact of advance directives as a means for increased consumer participation, with consumers reporting that their statements were ignored [25]. Clinicians reported varying levels of consumer involvement in the CTO process, with some stating it was ‘little or none’ , others that it was increased [51] and some reporting attempts to offer consumers choice and participation in decision-making [45]. To add to the complexity workers themselves were found to have inconsistent understandings about CTOs [23, 29].

Assessment of carer needs and input into care planning varied widely, with some carers choosing to “take a step back” and others reporting “being excluded from decision-making” (p. 1880) [29]. Some carers reported their involvement in care planning as infrequent, which was seen as contradictory given the high level of care they provided [42]. Others reported having an increased voice subsequent to the CTO process and feeling that their caring role was more recognised [36]. Interestingly, some carers reported increased involvement at the initiation of the CTO with less communication from mental health services over time, which was the opposite to reported consumer experiences of increased involvement with time [48, 54]. Issues related to confidentiality were cited as barriers to carers receiving information about their relative [28]. Overall, as consumer participation was reported to be low, increased involvement was recommended at all stages of the CTO process to enhance consumer empowerment [48]. Authors of a recent study found ‘CTOs were more successful when they were a carefully planned intervention [inclusive of the consumer and their family], rather than where they were made almost as a matter of course’ (p.91) [36]. Mfoafo-M’Carthy and Williams [5] went further and suggested mandated treatment could be discussed and presented as an intervention option under an advanced directive with individuals during a period of improved capacity. This approach however was on the proviso that the CTO was part of a more holistic care plan and approach. Currently, the provision of services to address consumers’ broader needs is not a statutory requirement of the CTO, and is dependent on the individual case manager [51], though Brophy et al. [44] suggest efforts should be made by case managers to address broader identified needs to meet consumer goals and redress the negative experience consumers often have of being on a CTO [43].

### Care co-ordination

Care co-ordination ‘involves continual communication with [consumers], their carers, and the various professionals and services…fundamental to care co-ordination is the … case manager’ (p.6) [4]. Case managers can have a significant impact on consumers, who can experience the support as either positive or negative with the potential to ‘either assist or obstruct recovery’ [45, 55]. Owens and Brophy [40] for example, found workers were not making sufficient efforts to manage the risk of recall to hospital or the distress experienced by consumers subsequent to this. Establishing good therapeutic relationships and family involvement are necessary to good care coordination [24]. The main findings under the theme of care-coordination relate to the therapeutic relationship and impact of coercion on this.

#### Impact of coercion on the therapeutic relationship

##### Dilemmas and challenges for workers

Various findings were reported regarding the impact of the CTO on the relationship between the case manager and consumer. Mullen at al. described it as an ‘apparent paradox that good therapeutic relations seemed to be required for a CTO to be effective’ and considered collaborative relationships integral to the success of a CTO (p. 542) [39]. Workers in this study spoke of needing to establish relationships based on trust and encouragement, aware that ‘rehabilitation’ could not be forced [39]. In the same study, highlighting the coercive aspect of CTOs, workers admitted to using threat of return to hospital if consumers were non-compliant with orders [7]. Brophy and McDermott viewed this dilemma as a daily compromise faced by case managers ‘between acting paternalistically, in what might be understood as the client’s best interests, and a competing requirement to respond to their expressed wishes’ (p. 158) [56]. Studies show workers are aware of the dilemma of wanting to support a person’s ‘right to self-determination while obtaining the benefits…possible with treatment adherence’ (p. 520) [6]. Lawn et al. [45] framed this as a moral dilemma experienced by staff, and found some staff more attuned to the impact of CTOs on consumers and the therapeutic relationship, and others less so. Moral interpretations were found to be made by workers and consumers regarding various issues encountered in the care planning space [45]. This included workers seeing consumers as ‘wilfully’ stopping medication and consumers reporting the need to overcome a ‘vice’ in order to be discharged from a CTO. These negative framings impacted upon both the care planning relationship (for example workers ‘punishing’ the consumer for not taking medication) and the consumers’ sense of self. Consumers learnt that to be ‘perceived as morally worthy’ , they had to ‘say the right thing’ [45]. To address this, Lawn et al. highlight the importance of worker empathy in engaging consumers on CTOs and the need for workers to consider ‘the relationship between what is done and how it is done’ (p. 15) [45]. Interestingly in another study, those workers who viewed CTOs as primarily coercive also reported discomfort in working with consumers on CTOs [50].

Workers recognised the importance of developing a therapeutic alliance with consumers, and reported on the stress that resulted from working in conditions that at times involved ‘hostility’ , ‘manipulation’ and ‘deceit’ [6], with one case manager describing their role as sometimes more aligned to correctional services than clinical treatment [50]. To redress the balance of power, workers have a responsibility to empower consumers by providing clear information about CTO processes and facilitating as much choice and involvement as possible in decision-making at all stages [35, 41, 48]. Workers acknowledged the ‘legal recognition’ of care that came with CTOs placing a greater responsibility on them to effectively engage consumers [36] as well as the challenges of effective engagement and the intensive nature of support required of person-centred care [48]. Brophy and McDermott [24] considered continuity of care important in providing quality care with this client group and suggested psychiatrists take a more central role as they were typically a more stable team member. In other studies however consumers reported more strained relationships with treating doctors, preferring to engage with case managers [35].

##### Relational quality

Consumer reports of the impact of the CTO on the relationship with their worker were varied, with some reporting no differences and others remaining angry towards workers [6]. Consumers reporting positive relationships with workers also appeared to have an overall positive experience of being on a CTO, and associated positive outcomes including improved mental health, support, relationships and occupational gains [27, 43, 55]. Relational aspects mentioned by consumers who reported positive rapport included workers who expressed concern, were helpful, supportive, didn’t view them as patients and with whom they met regularly [23, 35]. Lawn et al. [45] exploring the nuances of the therapeutic relationship between consumers and mental health workers, highlighted the complexity of developing trust within this dynamic. Interestingly, the authors found that whilst mental health workers had the expectation that consumers should trust and engage with them, as they had ‘good intentions’ and were ‘there to help’ , consumers experienced that they were not trusted themselves by mental health professionals. Steun et al. [57] also discussed the importance of developing reciprocal trusting relationships, with consumers reporting worker availability and support with everyday problems (such as housing, finances and social isolation) enhancing such relationships and positively impacting upon their experience of the ‘restrictive interventions’ of CTOs.

##### Negative impacts on consumers

Whilst some consumers reported a ‘blurred distinction between formal and informal coercion’ with treatment pressure a usual experience of mental health care (p.6) [43], others on CTOs experienced contact with mental health services to be more intrusive and coercive than the same contact had been experienced prior to the CTO [53, 58, 59]. The use of persuasion was found to be more common for those on CTOs and resulted in significantly higher levels of perceived coercion. Issues that negatively impacted upon establishing trusting relationships included lack of information from workers [48] and lack of involvement in decision-making, regularly reported as a lack of information and influence on medication [43, 53]. Consumers’ feelings of distrust towards workers was linked to the distress that resulted from the impact of CTOs on their liberty and rights, with interpersonal problems, including relationships with workers, linked to a sense of unhappiness [60]. Banks et al. [48] suggest the issue of choice is further complicated by the fact that consumers often retrospectively viewed restrictions in choice positively. Whilst studies reported increased acceptance of CTOs by consumers over time, often related to positive gains [6, 36, 55], even those considered to be ‘generally favourable about the CTO’ still identified negative aspects including feeling restricted, stigmatised, untrusted by mental health workers and a lack of control [7, 61]. Three quantitative studies explored consumers’ perceptions of coercion. Though overall consumers on CTOs reported experiencing greater coercion then voluntary consumers and less satisfaction with care [58, 60], some consumers found that over time service pressure could be helpful [26]. McKenna et al. state that a small level of coercion may have a positive impact on therapeutic outcome, though warn that ‘the correct amount of coercion is titrated and then sustained’ (p.155) [58].

##### Mixed consumer experience

Of those papers reporting on consumer experience of CTOs in general, the majority reported mixed experiences, with a similar number of findings referencing positive or negative experiences. This variation highlights the complexity and individual response consumers have to being on a CTO. One paper comparing views of consumers from different ethnic backgrounds (Maori and Non-Maori) found few differences [62]. Dawson et al. [63] described some consumers as ‘volunteers for compulsion’ , though acknowledged that even those ‘voluntary’ consumers often had a complicated relationship to the CTO with variation in experiences over time. CTOs were seen as favourable to most consumers over hospital stays and often seemed to account for their positive view [28]. Reported benefits included increased support, a sense of security, improved access to services and hospital avoidance [6, 29, 48, 53, 54] with some consumers viewing CTOs ‘as a transitional step from a chaotic to a more stable life’ (p.366) [35]. One study found no association for consumers between being on a CTO and recovery beliefs [64], however negative impacts for consumers on CTOs were significant and included, ‘side-effects of enforced medication…an enduring sense of stigma; restrictions on place of residence …limited social and work opportunities; the feeling that others made key decisions about their lives; and not getting better, merely existing’ (p.822) [28]. Consumers likened their experience of treatment by others to that of a child or criminal [23, 29], with some referring to their own home as an institution in the community [53].

#### Impact of support type and frequency

Reported support type and frequency varied. Some consumers reported frequent (daily) contact and support with an emotional focus, practical tasks and social engagement, with this type of support related to positive care experiences [49]. Others reported less frequent contact and dissatisfaction when the focus was primarily on medication [51]. Given the high level of needs typical to consumers on CTOs, it was surprising that there was little evidence of use of specialised interventions [24]. Though consumers on CTOs were often unemployed and living in difficult conditions, only a minority were receiving assertive care or input from psychosocial supports [40]. Brophy et al. [24] stressed the need for workers to provide psychological, social and occupational interventions and avoid over-focussing on medication. Other interventions proposed to reduce the coercive impact of CTOs and promote consumer participation included the use of advanced directives, shared decision-making and increased access to independent advocates [25, 50].

#### Limited linking with broader services

Though consumers on CTOs typically have complex needs that require linking with various services there was minimal reference to this in the included studies. Light et al. found GPs had a key role with consumers on CTOs as they addressed the persons’ broader health needs, provided mandated psychiatric treatment (depot administration) and ‘enhanced patient care by… building strong therapeutic relationships and ‘normalising’ treatment’ (p.487) [65]. Interestingly the authors found minimal reference to GPs in CTO literature and policy. Conversely, references to engaging with families were made in the majority of studies, with family members often study participants. Family members were aware of potential dilemmas and tensions that came with CTO use including differing opinions between them and their relative [42, 54], though often reported positive benefits of CTOs, such as increased stability for their relatives and increased connection with services and support for them and their family member [6, 7, 48]. Whilst the CTO gave carers evidence that their relatives illness was being taken seriously by services, they remained the primary caregiver with the major responsibility for care. Family members requested increased inclusion from services as they were the frontline support when the system failed to adequately address their relatives’ needs [42]. Clarity around who to contact, and how to request an emergency review, reassured carers [36].

### Case closure- discharge from CTO

An individual’s autonomy and rights are impacted upon by a CTO, and the aim should be that the person resumes personal control and does not require the CTO [44], with workers having a responsibility to support consumers towards discharge from treatment orders [39]. The findings indicated significant confusion around when a consumer should be discharged from a CTO.

#### Lack of definite criteria for discharge

The majority of qualitative studies did not directly explore discharge. Workers had difficulty identifying optimal indicators for discharging consumers from orders, with differing opinions reported in the multidisciplinary team and factors other than current presentation impacting upon the decision (e.g. the consumers risk profile and workers previous experience of discharge) [7]. Factors that facilitated discharge included sustained compliance, clinical improvement, reduced risk, greater stability and insight, taking responsibility for treatment and engaging with the treating team [7, 31, 33, 34, 39]. Brophy and Ring [51] found medication compliance and improved insight were linked by workers and the primary basis for discharge, though interestingly, Rugkasa et al. [26], reporting on quantitative data, found no changes in consumer insight and attitudes to treatment (including adherence to medication) between consumers on CTOs and consumers not on CTOs. Dawson et al. state the lack of ‘definite criterion of success in compulsory community care’ results in ‘the dilemma of discharge’ and queried if long term use of CTOs resulting in hospital avoidance should be considered ‘a successful or an unnecessary (and therefore overly coercive) form of intervention’ (p.250) [63].

#### Impact on consumers

Lack of clarity regarding discharge impacted on consumers who reported discharge as difficult to obtain [35]. Additionally, lack of certainty about the duration of CTOs was experienced negatively by consumers [43, 66] with some reporting becoming dependant on the mental health system subsequent to being on orders for prolonged periods [61]. Consumers reported reasons for compliance with CTOs included avoiding hospital, to prevent another CTO, fear of relapse, family pressure and seeking to gain greater stability [6, 28, 36, 67]. Based on the lack of clarity regarding discharge, workers need to be more transparent with consumers regarding processes and conditions of discharge [7].

### Benefits of case management

#### Service utilisation

Case management aims to reduce the need for service contacts, particularly hospital utilisation [4]. Dawson et al. [63] reviewed studies claiming CTOs reduced the need for hospilitisation however found they had not sufficiently accounted for changes in mental health services, introduction of more effective medications or interventions received in the community. For the purpose of this review, given consumers on CTOs are forced to receive treatment, the data was explored regarding CTO impact on facilitating service access according to individual need.

##### CTO impact on service access and referrals

In summary, studies often stated CTOs facilitated access to mental health professionals and services, with easy access reported as benefits of CTOs by consumers and their carers [49] [41]. Increased access to accommodation services was also reported, with accommodation staff reporting that they felt more supported by mental health workers when CTOs were in place [39]. Conversely, some consumers reported that the negative impact of being on a CTO would mean that they would avoid seeking help in the future [55]. In other papers, the small numbers of consumers receiving assertive and intensive psychosocial support as well as limited resources in rural areas was highlighted, indicating CTOs do not always enhance access to needed services and supports [7, 40]. It was often unclear in the studies if this was a consequence of lack of infra-structure and resources or poor referral and linking.

#### Health outcomes

Case management has been shown to have a positive impact on health outcomes, though it is acknowledged that measuring such outcomes is complex. Health outcomes include: ‘quality of life, independence, functionality and general well-being’ (p. 13) [4]. For this theme, data relating to consumer, worker and family perspectives on the usefulness of CTOs in enhancing the above domains for the consumer was explored.

#### Balancing costs and gains for the consumer

##### Consumer perspective

Consumer perspectives on the usefulness of CTOs varied. Some consumers considered CTOs to be a barrier to their recovery and negatively impacting on their sense of self-worth, self-direction and relations to others in the broader community [49, 53, 61]. Being on a CTO was experienced as humiliating, embarrassing and more stigmatising than having a diagnosis of mental illness [41, 51, 53]. Others reported improved self-worth and a sense of empowerment linked to functional gains, improved relationships and success in finding employment [7, 55]. Interestingly, when positive gains were reported, there was a lack of consistency regarding what facilitated improvements, with some reporting medication adherence and others increased support as primary facilitators [23]. Furthermore, some family members reported that gains such as employment were a result of the individuals own efforts rather than service support [25].

##### Worker and family perspective

Though some workers reported observing positive gains including, risk reduction, relapse detection, hospital prevention and housing stability, they challenged whether being on a CTO enhanced social inclusion, reporting a lack of gain in meaningful occupation and no positive changes in stigma or discrimination [30]. Workers were generally reluctant to attribute positive changes to the CTO alone [29, 51]. Similarly, family members thought increased and regular engagement with workers, rather than the powers of the CTO, was what resulted in improved compliance [54]. Furthermore though family members often reported improvements in their relatives social and occupational functioning, they were critical when the focus of care was symptom amelioration with medication, with one family member describing such care resulting in their child being ‘simply “contained” at home rather than hospital’ (p.1880) [29]. Positive impacts for family members included improved family relations, a sense of relief, increased safety [54] and feeling empowered and supported when actively involved in the CTO process [42].

## Broader issues that support effective case management

Various broader issues impact upon the effectiveness of case management and consumer outcomes. These include resources, manageable caseloads, effective linking with stakeholders from different service sectors and continuity of care [4]. These broader service issues were referred to in several of the included papers. Limited resources and service availability were reported to impact on decisions around CTO use as well as result in increased use of CTOs to facilitate early discharge from inpatient services [31, 37, 48] and access to limited inpatient beds [29, 31]. Psychiatrists reported high caseloads, insufficient time available to spend with consumers and reduced service options in rural areas [7].

Few studies reported on links with a broad range of stakeholders. Light et al. [65], exploring links with primary care, found a lack of integration between primary care and mental health services, though reported some instances where systems were established to enhance shared care between GPs and mental health services. Gibbs et al. [28] reported a lack of linking of mental health teams with supported accommodation services. Even within mental health services, workers referred to a ‘silo-mentality’ with poor communication and poor linking between inpatient and outpatient services negatively impacting upon consumers [31, 37, 40]. Lack of continuity of care was also found to lead to increased tensions for workers, for example when workers were required to adhere to CTO conditions put in place by others [48, 67].

## Discussion

The studies included in this review provide rich data that relates to consumers, carers and mental health workers perspectives and experiences of care coordination and care planning for consumers on CTOs in community mental health care settings across a range of countries. Many of the issues also relate more broadly to those individuals whom have a mental illness and may present with complex needs. Models of case management differ in terms of staffing, caseload number, contact frequency, length and availability of service and treatment options and responsibilities [68]. Understanding the various issues that impact upon the implementation of CTOs, including service delivery models and resources is important in order to inform best practice [24].

A key finding of this review was the lack of connection between CTO goals (which are service driven) and recovery goals (which are consumer driven), with minimal reference made to care plans documenting the care planning process. Furthermore given the lack of consumer input and knowledge of care plans, it was difficult to substantiate consumer involvement [40]. Several papers identified the need to link CTO goals to treatment and consumer goals [5, 24, 30]. Such linking would promote collaborative care planning, facilitate care that is person-centred (and not overly focussed on service goals of medication compliance) and promote service responsibility and support with the consumers’ broader goals, including discharge from orders. Even linking CTO purpose to treatment goals would enhance worker accountability.

Lack of clarity of the purpose of CTOs further complicates linking CTO and consumer goals. Kisely and O’Reilly question if the purpose of the CTO is to ‘reduce revolving-door admissions, provide a less restrictive alternative to involuntary admission, prevent violence by people with severe mental illness, or increase stability and promote recovery’ (p.415) [69]. The CTO purpose will impact upon both the focus of interventions and expected outcomes including ‘hospital use, perceived coercion, violent acts and quality of life’ (p.415) [69]. This is important given the lack of clarity regarding discharge from orders. CTO processes of assessment, review and discharge from orders are incorporated into the case management role. In Australia, mental health tribunal reviews are conducted 12 monthly. In addition to these formal reviews, care coordinators are required to regularly review an individual’s care (typically 3 monthly). This multidisciplinary review process provides regular opportunities to review changes against both CTO and individual recovery goals, ensure required supports are in place, prompt consideration of discharge and ensure consumers are not left languishing on CTOs. There was little evidence of regular reviews and early discharge from CTOs in the included studies and only three studies that recruited all key stakeholders involved in the care planning relationship. Further exploration of how case management can better incorporate and manage issues related to CTOs is warranted.

A core component of care planning is identifying and implementing relevant evidence based interventions [4], yet none of the included studies specifically examined the usefulness of focussed interventions. Studies exploring the use of crisis planning and advanced directives identified in the search specifically excluded individuals on CTOs [70, 71]. Increased stakeholder participation (of workers, consumers and carers) during mental health tribunals was recommended to enhance decision-making related to CTOs [24], with a particular focus on promoting consumer participation in early stages of CTO implementation [48]. Shared decision-making (SDM) is a core concept in care-planning and builds on person centeredness by promoting mutual expertise and determining the individuals ‘preferred role in the decision-making process’ [19]. In mental health care, SDM is often referred to in the context of supporting consumers’ to make informed decisions related to medication [72, 73]. A recent randomised trial of a patient decision aid for individuals with PTSD, reported increased consumer knowledge of their condition and reduced conflict regarding treatment choice [74]. Recent studies aiming to enhance medication compliance of consumers with mental health problems have explored the use of peer workers [75], motivational interviewing [76] and treatment adherence therapy [77] with results indicating some success. Given consumer dissatisfaction with their level of involvement in care planning, decisions related to the CTO process, and over focus on medication, focussed interventions to enhance decision-making and medication compliance for consumers’ on CTOs are important areas to further explore.

Various recommendations for practice were made in the included studies. Mfoafa-M’Carthy and Shera considered ‘CTOs should be a voluntary contractually based community treatment option of last resort’ (p.76) [68] and suggested providing less coercive support options for people with serious mental illness, including intensive case management and use of advanced directives to increase collaborative care planning. Brophy and McDermott [24] took a more pragmatic approach, and acknowledging CTOs were part of current mental health care, sought key stakeholders perspectives on how to “do CTOs well”. Identified principles of good practice included: taking a human rights perspective (being aware of peoples’ right to self-determination); being transparent regarding CTO goals and purposes and linking these to treatment goals; providing quality services (including continuity of care and evidence-based interventions); facilitating involvement of consumers and their carers’ in the CTO process and development and use of direct practice skills (including linking with support staff and development of advanced interpersonal skills) [24]. Similarly, Lehssier et al. [19] emphasised the need for case managers to have advanced practitioner skills, such as SDM and motivational interviewing.

Stuen et al. [43] found an assertive engagement approach with psychosocial interventions was as beneficial as the CTO in engaging ‘reluctant consumers’ in treatment. Similarly, Churchill et al. [78] conducted a comprehensive review of research of experiences of CTO use internationally and recommended exploring the ‘potential therapeutic gains [that] might be better delivered by enhancing the quality and assertiveness of community treatment for high risk patients’ through, for example, ACT’. Core elements of ACT include ‘assertive engagement, small caseloads [and] focus on supporting broad life domains’ (p.11) [43]. Whilst this approach has clear benefits in engaging consumers around their identified goals, referral to services that are able to provide psychosocial support is more widely available and should be considered more often than was evident in the studies [24]. In addition to linking with broader services, the recovery literature recommends a focus on linking consumers with their personal and community resources to support everyday connections and reduce dependence on health services [79]. There was little reference of such linking in the included studies other than with consumers’ families, and a few reports of links with GPs and accommodation services [39, 65].

Most papers made reference to the coercive nature of CTOs and potential impact on the therapeutic relationship, which is key to effective case management. Some authors whom have published extensively on involuntary psychiatric treatment have backgrounds in socio-legal research and/or social work. Brophy and McDermott for example used critical social work theory to explore best practice with individuals on CTOs, and highlighted the role this theory has in ‘encourag[ing] social workers to be mindful of the imbalance of power that is inherent in all social work practice’ (p.74) [24]. In clinical practice, case managers have varied professional backgrounds and may be less sensitive to some of the issues of care and control inherent in the care relationship, as these issues may not be addressed in undergraduate training. Lawn et al. highlight the potential for the relationship between mental health workers and consumers to ‘either assist or obstruct recovery’ (p.14) [45]. Key components of the therapeutic relationship in the context of forced treatment included empathic skills and trusting relationships [45]. Consumers who trust health services and workers have better clinical outcomes and report increased positive care experiences [80]. Trusting relationships are considered ‘a prerequisite to the negotiation of reciprocal agreements [which], in turn, lead to patient-centred care’ (p.886) [81]. ‘[Worker] characteristics that have been shown to encourage patient trust [include] ability (also termed competence), benevolence, integrity, respect, and honesty’ (p.7) [80]. The role these relational factors have in facilitating therapeutic alliance has a longstanding and robust evidence base, however Davidson and Chan [82] warn that it should not be assumed that such skills are already being practiced, and that empathy skills should be developed and maintained with targeted training, reflection and supervision [45, 82].

### Limitations

Appraisal and data extraction was conducted by only one author, though opinion was sought from a 2nd reviewer to clarify studies for inclusion. A limitation of qualitative studies is a lack of generalisability to broader contexts, though the integrative review method of synthesising data from different studies conducted in different locations helps address this. Quantitative studies were not reported in detail, with the decision made to utilise these data to augment the more in-depth qualitative findings in order to best answer the research question.

## Conclusion

The effectiveness of case management will be influenced by various factors, including the quality of relationship established between consumers and workers and the type of support offered to consumers. These factors are interrelated and dependent on good assessment of needs, as well as resources available in the community (inclusive of housing, financial security, substance abuse programs and supports to facilitate social connections) [38, 68]. As Davidson [83] points out, ‘personal choice plays a very limited role, … when the person has very limited, if any, choices to begin with’ (p.366) [83]. CTO legislation, service delivery models and resource availability all impact upon the implementation of CTOs and need to be considered when exploring best practices [24] {Brophy, 2013 #807; }. Changes at the level of clinical practice however can still positively impact on consumers’ experiences of CTOs. The conflicting processes of reciprocity, which involves mutual trust, and authority in current mental health practice needs to be recognised [81] with person-centred approaches requiring core practitioner and communication skills including empathy, trust and hope [19]. Workers should aim to engage in the care planning process in ways that enhance consumer experience (increased consumer involvement and addressing identified consumer needs) whilst being sensitive to the ‘control and care’ dynamic of the relationship.

## Additional files

Additional file 1: Search strategy. (DOCX 22 kb)
Additional file 2: Details of included studies. (DOC 142 kb)

## Abbreviation

CTO: Community treatment order
