# Supplementary material for: Care planning for consumers on community treatment orders: an integrative literature review
Source: BMC Psychiatry. 2016 Nov 10;16:394. doi: 10.1186/s12888-016-1107-z (PMC5105250; doi:10.1186/s12888-016-1107-z)
Supplement: Additional file 1: — Search strategy. (DOCX 22 kb) [file 12888_2016_1107_MOESM1_ESM.docx]

# Additional file 1: Search strategy

# Community Treatment Orders – Literature search

## Databases searched:

## PsycINFO (Ovid)

- Medline (Ovid)
- PubMed (non-Medline content only)
- Scopus
- CINAHL
- ProQuest (Social Sciences and Health subsets only)

Total number of citations before duplicates removed n = 7459

Total number of citations after duplicates removed n = 4283

## PsycINFO 1806 to September Week 1 2015

Search executed 9/9/15

| **#** | **Searches** | **Results** |
| --- | --- | --- |
| 1 | outpatient commitment/ | 185 |
| 2 | (community treatment order* or CTO* or outpatient commitment or outpatient treatment or AOT).tw. | 4387 |
| 3 | (community or outpatient or out-patient).tw. | 234174 |
| 4 | ((involuntary or order* or coerc* or compulsory or commitment or legal or law or mandate*) adj3 treatment).tw. | 4540 |
| 5 | 3 and 4 | 1018 |
| 6 | 1 or 2 or 5 | 4989 |
| 7 | chronic mental illness/ or chronic psychosis/ | 1660 |
| 8 | exp psychosis/ or exp schizophrenia/ or paranoid schizophrenia/ | 100475 |
| 9 | mental disorders/ or chronic mental illness/ or personality disorders/ or schizoaffective disorder/ or abnormal psychology/ or borderline states/ or comorbidity/ or psychiatric patients/ or schizophrenogenic family/ or suicide/ or treatment resistant disorders/ | 157705 |
| 10 | (mental* or psychos* or psychot* or psychiatr* or schizo* or personality disorder* or suicid*).tw. | 807207 |
| 11 | or/7-10 | 828710 |
| 12 | 6 and 11 | 3067 |
| 13 | limit 12 to english language | 2686 |
| 14 | limit 13 to yr="2000 - 2015" | 1667 |

## Ovid MEDLINE(R) In-Process & Other Non-Indexed Citations and Ovid MEDLINE(R) 1946 to Present

Search executed 9/9/15

| **#** | **Searches** | **Results** |
| --- | --- | --- |
| 1 | "Community Mental Health Services"/ | 17527 |
| 2 | lj.fs. or mandated programs/ or coercion/ or commitment of mentally ill/ | 237744 |
| 3 | 1 and 2 | 1174 |
| 4 | (community treatment order* or CTO* or outpatient commitment or outpatient treatment or AOT).tw. | 8162 |
| 5 | (community or outpatient or out-patient).tw. | 451689 |
| 6 | ((involuntary or order* or coerc* or compulsory or commitment or legal or law or mandate*) adj3 treatment).tw. | 7553 |
| 7 | 5 and 6 | 812 |
| 8 | 3 or 4 or 7 | 9594 |
| 9 | mental disorders/ or exp mood disorders/ or exp personality disorders/ or exp "schizophrenia and disorders with psychotic features"/ | 383743 |
| 10 | Mentally Ill Persons/ | 5624 |
| 11 | suicide/ or suicidal ideation/ or suicide, attempted/ | 48677 |
| 12 | (mental* or psychos* or psychot* or psychiatric or schizo* or personality disorder* or suicid*).tw. | 666577 |
| 13 | or/9-12 | 824893 |
| 14 | 8 and 13 | 2736 |
| 15 | limit 14 to (english language and yr="2000 - 2015") | 1451 |

## PubMed

Searched 9/9/15

N=42

(community treatment order*[tiab] OR CTO*[tiab] OR ((community treatment[tiab] OR community care treatment[tiab] OR outpatient treatment[tiab] OR out-patient treatment[tiab] OR AOT or outpatient commitment[tiab] OR out-patient commitment[tiab]) AND (involuntary[tiab] OR order*[tiab] OR coerc*[tiab] OR compulsory[tiab] OR commitment[tiab] OR legal[tiab] OR law[tiab] OR mandate*)) AND (mental*[tiab] OR psychos*[tiab] OR psychot*[tiab] OR psychiatric[tiab] OR schizoid*[tiab] OR schizophreni*[tiab] OR personality disorder*[tiab] OR suicid*[tiab]) AND English[la] AND 2000:2015[dp]) NOT Medline[sb]

## CINAHL

9/9/15

| **#** | **Query** | **Limiters/Expanders** | **Results** |
| --- | --- | --- | --- |
| S1 | (MH "Community Mental Health Services") OR (MH "Community Mental Health Nursing") OR (MH "Social Work, Psychiatric") | Search modes - Boolean/Phrase | 8,310 |
| S2 | ( (MH "Involuntary Commitment") OR (MH "Medical Orders") OR (MH "Patient Compliance") OR (MH "Coercion") ) OR MW legislation | Search modes - Boolean/Phrase | 119,727 |
| S3 | S1 AND S2 | Search modes - Boolean/Phrase | 687 |
| S4 | TI ( "community treatment order*" OR CTO* ) OR AB ( "community treatment order*" OR CTO* OR "outpatient commitment" or "outpatient treatment" or AOT ) | Search modes - Boolean/Phrase | 1,051 |
| S5 | TI ( community OR outpatient OR "out-patient" ) OR AB ( community OR outpatient OR "out-patient" ) | Search modes - Boolean/Phrase | 133,313 |
| S6 | TI ( ((involuntary OR order* OR coerc* OR compulsory OR commitment OR legal OR law OR mandate*) N3 treatment) ) OR AB ( ((involuntary OR order* OR coerc* OR compulsory OR commitment OR legal OR law OR mandate*) N3 treatment) ) | Search modes - Boolean/Phrase | 1,842 |
| S7 | S5 AND S6 | Search modes - Boolean/Phrase | 375 |
| S8 | S3 OR S4 OR S7 | Search modes - Boolean/Phrase | 1,860 |
| S9 | (MH "Psychiatric Patients") OR ( (MH "Mental Disorders") OR (MH "Behavioral and Mental Disorders") OR (MH "Mental Disorders, Chronic") OR (MH "Psychotic Disorders") OR (MH "Affective Disorders, Psychotic") OR (MH "Bipolar Disorder+") OR (MH "Paranoid Disorders") OR (MH "Postpartum Psychosis") OR (MH "Schizoaffective Disorder") OR (MH "Schizophrenia") OR (MH "Personality Disorders+") ) OR ( (MH "Suicide") OR (MH "Suicide, Attempted") OR (MH "Suicidal Ideation") ) | Search modes - Boolean/Phrase | 68,183 |
| S10 | TI (mental* OR psychos* OR psychot* OR psychiatr* OR schizo* OR "personality disorder*" OR suicid*) OR AB (mental* OR psychos* OR psychot* OR psychiatr* OR schizo* OR "personality disorder*" OR suicid* ) | Search modes - Boolean/Phrase | 142,360 |
| S11 | S9 OR S10 | Search modes - Boolean/Phrase | 165,165 |
| S12 | S8 AND S11 | Limiters - Published Date: 20000101-20151231; English Language  Search modes - Boolean/Phrase | 713 |

## Scopus

Searched 9/9/15

N=1991

( TITLE-ABS-KEY ( ( "community treatment order*"  OR  cto*  OR  "outpatient commitment"  OR  "outpatient treatment"  OR  aot  OR  ( ( community  OR  outpatient  OR  "out-patient" )  AND  ( involuntary  OR  order*  OR  coerc*  OR  compulsory  OR  commitment  OR  legal  OR  law  OR  mandate* )  W/3  treatment ) ) )  AND  SUBJAREA ( mult  OR  medi  OR  nurs  OR  vete  OR  dent  OR  heal  OR  mult  OR  arts  OR  busi  OR  deci  OR  econ  OR  psyc  OR  soci )  AND  PUBYEAR  >  1999  AND  PUBYEAR  <  2016 )  AND  ( TITLE-ABS-KEY ( mental*  OR  psychos*  OR  psychot*  OR  psychiatr*  OR  schizo*  OR  "personality disorder*"  OR  suicid* )  AND  SUBJAREA ( mult  OR  medi  OR  nurs  OR  vete  OR  dent  OR  heal  OR  mult  OR  arts  OR  busi  OR  deci  OR  econ  OR  psyc  OR  soci )  AND  PUBYEAR  >  1999  AND  PUBYEAR  <  2016 )  AND  ( LIMIT-TO ( LANGUAGE ,  "English" ) )  AND  ( LIMIT-TO ( DOCTYPE ,  "ar" )  OR  LIMIT-TO ( DOCTYPE ,  "re" )  OR  LIMIT-TO ( DOCTYPE ,  "cp" )  OR  LIMIT-TO ( DOCTYPE ,  "ip" ) )

## ProQuest

Searched 9/9/15

N=1595

all("community treatment order*" OR CTO* OR "outpatient commitment" OR "outpatient treatment" OR aot OR ((community OR outpatient OR "out-patient") AND ((involuntary OR order* OR coerc* OR compulsory OR commitment OR legal OR law OR mandate*) NEAR/3 treatment))) AND all(mental* OR psychos* OR psychot* OR psychiatric OR schizo* OR "personality disorder*" OR suicid*)

Limited to English, Scholarly journals, and January 2000 to September 2015
