# Supplementary material for: Care planning for consumers on community treatment orders: an integrative literature review
Source: BMC Psychiatry. 2016 Nov 10;16:394. doi: 10.1186/s12888-016-1107-z (PMC5105250; doi:10.1186/s12888-016-1107-z)
Supplement: Additional file 2: — Details of included studies. (DOC 142 kb) [file 12888_2016_1107_MOESM2_ESM.doc]

| **Quantitative studies** | | | | | |
| --- | --- | --- | --- | --- | --- |
| **Author and Date** | **Year** | **Aim/Objective** | **Context/Setting** | **Methods and Methodology** | **Findings related to the review** |
| DeRidder, R.,  Molodynski, A.,  Manning, C.,  McCusker, P.,  Rugka˚sa, J.  Community treatment orders in the UK 5 years on: a repeat national survey of psychiatrists | 2016 | To establish psychiatrists experiences and current  opinions of using CTOs and to compare findings with a previous survey conducted in 2010. | UK | Survey. Statistical and descriptive analysis conducted. | The opinions of psychiatrists in the UK have not changed since 2010 despite recent evidence questioning the effectiveness of CTOs. This was viewed as concerning. Clinical factors (the need for engagement and treatment adherence, and the achievement of adherence and improved insight) remain the most important considerations in initiating and discharging a CTO. |
| Rugkasa, J.,  Molodynski, A.,  Yeeles, K.,  Montes, M.,  Visser, C.,  Burns, T. Community treatment orders: Clinical and social outcomes, and a subgroup analysis from the OCTET RCT | 2015 | To test the effect of community compulsion on wider clinical and social outcomes and on patients' experiences of services and the use of treatment pressure and explore differential effects in different groups of patients.  Reporting on OCTET. | UK, 336 eligible patients were randomised, with data collected for 333 patients. Eligible participants were patients in adult services  (18–65 years) with a diagnosis of psychosis,  considered appropriate for CTO by their clinical  team and able to give informed consent | Non-blinded RCT of CTO effectiveness.  Hospitalisation data and data on medication from medical records.  Symptoms, social functioning and patient-rated outcomes from interviews at baseline, 6 and 12 months. | Compelling patients to adhere to treatment does not have benefits for consumers on a wide range of clinical and social outcomes. One finding (of difference) was those in CTO arm of trial showed a smaller increase over time than controls in their agreement that pressure in services could be helpful. |
| Newton-Howes, G., Banks, D. The subjective experience of community treatment orders: Patients' views and clinical correlations | 2014 | To assess patients’ perspectives of CTOs in order to identify correlations between clinical and demographic variables and a positive experience of CTOs. | NZ  79 adults subject to a CTO for at least 6 months (on May 2010) and anyone subject to a CTO for a min of 6 months prior to this date and cared for a secondary services. | Questionnaires (self-report measures). | 53% felt they were on balance better off when treated informally in the community; patients described greater coercion and less satisfaction with care when subject to a CTO- these factors and being in employment identified patients whom felt harmed by CTOs 61% of the time. |
| Newton-Howes, G., Lacey, CJ.,  Banks, D. Community treatment orders: The experiences of non-Maori and Maori within mainstream and Maori mental health services | 2014 | To compare views of Maori consumers and non-Maori consumers about CTOs. | NZ  79 adults subject to a CTO for at least 6 months (on May 2010) and anyone subject to a CTO for a min of 6 months prior to this date and cared for a secondary services. | Questionnaires (self-report measures). | There were few differences in views of Maori compared to non-Maori consumers and no difference in views of Maori consumers cared for by mainstream compared to culturally specialist MHS. |
| Newton-Howes, G. A factor analysis of patients' views of compulsory community treatment orders: The factors associated with detention | 2013 | To examine the views of patients currently or previously detained under a CTO. | NZ  79 adults subject to a CTO for at least 6 months (on May 2010) and anyone subject to a CTO for a min of 6 months prior to this date and cared for secondary services. | Self-report measures were used to identify patients’ views on compulsory treatment. | Three overlapping factors were identified: interpersonal difficulties; intrapsychic threat and a safety factor associated with detention. |
| Fahy, GM.,  Javaid, S.,  Best, J. Supervised community treatment: Patient perspectives in two Merseyside mental health teams | 2013 | To explore patient perspectives subject to CTOs. | UK, NHS, 2 mental health teams.  17 patients from and an early intervention team and assertive outreach teams currently under Supervised Community Treatment, from point of hospital d/c. | Survey.  Structured interview with Likert scale. | Majority of patients believed TO facilitated earlier hospital d/c but felt uninvolved in the process. A significant proportion lacked motivation or ability to understand verbal and written info pertaining to legal rights at the time it was given. All felt they must strictly abide by conditions to remain in the community. There was a lack of knowledge of harm criteria and ability for recall. |
| Coyle, D.,  Macpherson, R.,  Foy, C.,  Molodynski, A.,  Biju, M.,  Hayes, J. Compulsion in the community: Mental health professionals' views and experiences of CTOs | 2013 | To explore the views and experiences of MH professionals (including psychiatrists) re the use of CTOs. | UK, NHS, Oxford.  288 surveys completed (response rate of 48%). | Surveys. | 48 (83%) psychiatrists and 142 (67%) MH professionals were in favour of CTOs.  Decision-making regarding CTOs was overwhelmingly clinically orientated for all professional groups. There were significant differences in views between groups re. effects of bureaucracy, infringement of human rights and coercion.  MDT involvement is crucial in decisions regarding CTOs and may protect against poor practice. Further training and support for staff is needed. |
| Manning, C.,  Molodynski, A.,  Rugkasa, J.,  Dawson, J.,  Burns, T, Community treatment orders in England and Wales: national survey of clinicians views and use | 2011 | To ascertain views and experiences of psychiatrists in England and Wales of CTOs. | UK, Wales.  566 Psychiatrists responded (29% response rate) | Survey. | Respondents were generally positive, reported decision-making regarding compulsion was based largely on clinical grounds.  Authors call for MDT input into DM. |
| Patterson, T.,  Mullen, R.,  Gale, C.,  Gray, A. Compulsory community treatment and patients' perception of recovery in schizophrenia | 2011 | To examine whether patients perceptions of recovery differed for those receiving treatment under a CTO compared to those who were not. | NZ, Dunedin.  86 participants with a diagnosis of Schizophrenia or schizoaffective disorder. | Interview with battery of measures covering indices of mental & physical health, quality of life, insight, ethnicity and recovery. | There was no association between being under a CTO and recovery beliefs. The majority of patients (irrespective of if they were on an order) reported recovery as possible and ½ reported they were in recovery. |
| Christy, A., Petrila, J. Involuntary outpatient commitment in Florida: Case information and provider experience and opinions | 2009 | To examine key characteristics of the first 50 IOCs in Florida. To obtain the experiences and opinions of Florida Mental health professionals about IOC, including incentives and disincentives for using the statute. | Florida, USA. 498 licensed mental health professionals. | Online survey. | Respondents indicated various issues had reduced the use of IOC including difficulties in applying the statute, inadequate clinical resources and scepticism regarding the effect of IOCs on positive clinical outcomes. |
| Rolfe, T., Sheehan, B., Davidson, R. Are consumers on community treatment orders informed of their legal and human rights? A West Australian study | 2008 | To collect information from consumers who were currently or had recently been on CTOs as to their perceptions of whether they had been fully informed of their rights under the West Australian Mental health Act. | Western Australia. 86 consumer participants with experience of being on a CTO in the area of the study. | Postal survey. | Mental health clinicians need to make significant improvements in providing information to consumers. This may impact positively on consumer engagement and therapeutic relationships and lead to improved health outcomes and CTO compliance. |
| McKenna, BG., Simpson, AIF., Coverdale, JH. Outpatient commitment and coercion in New Zealand: A matched comparison study | 2006 | To determine the level of coercion perceived by those under outpatient commitment in New Zealand. | NZ, Auckland. 69 consumers under OC and 69 matched consumers who were voluntary. | A cross-sectional comparative study. Face: face structured interviews. | Though the level of coercion for involuntary outpatients was relatively low, it was significantly higher than that experienced by voluntary outpatients. Emotional responses of the consumer impacted upon their perception of coercion and the use of persuasion during treatment resulted in higher levels of perceived coercion. |
| Greenberg, D., Mazar, J., Brom, D., Barel, YC.  Involuntary Outpatient Commitment: A naturalistic study of its use and a consumer survey at one community mental health center in Israel | 2005 | To evaluate the effectiveness of IOC as a means of ensuring continuing psychiatric treatment, and reducing hospitalizations.  To ascertain patients and psychiatrists views on the IOC and its effect on their relationship. | Israel. 13 patients and 17 psychiatrists were interviewed. | Survey conducted via interview. | Majority of patients perceived the commitment in negative terms, although not all thought it had a negative impact on their relationship with the psychiatrist. Psychiatrists were often sensitive to the patient’s wishes, not all considered they were providing optimum treatment and the involuntary aspect of care was not always pursued. |
| Romans, S.,  Dawson, J.,  Mullen, R.,  Gibbs, A. How mental health clinicians view community treatment orders: A national New Zealand survey | 2004 | To determine New Zealand mental health clinicians’ views about community treatment orders, indications for their use, their benefits, problems and impact on patients and therapeutic relationships. | New Zealand. Psychiatrists (n= 202) and community MHPs (n=82), largest group nurses (n=35). | Survey  A national survey of New Zealand psychiatrists and regional survey of  community mental health professionals for comparison. | Majority of psychiatrists prefer to have CTOs an option. Consider they are used properly in most cases, can enhance priority for care, provide structure for treatment, support continuing contact and produce a period of stability allowing other therapeutic changes. They consider CTOs can harm therapeutic relationships, especially in the short term, but when used appropriately overall benefits outweigh coercive impact. MHPs surveyed had similar views with a minority of clinicians not supporting use. |
| Dawson, J.,  Romans, S. Uses of community treatment orders in New Zealand: Early findings | 2001 | To assess the uses of Community Treatment Orders (CommTOs) in New Zealand. | New Zealand. | Retrospective study of patients’ records of mental health legislation and a survey of psychiatrists. | There was a high level of agreement amongst psychiatrists that, when used appropriately, the benefits of CommTOs outweigh their coercive impact on patients. The most strongly supported indicator for use was the promotion of medication compliance. |
|  | | | | | |
| **Qualitative studies** | | | | | |
| **Author and Date** | **Year** | **Aim/Objective** | **Context/Setting** | **Methods and Methodology** | **Findings related to the review** |
| Banks, LC.,  Stroud, J.,  Doughty, K. CTOs: exploring the paradox of personalisation under compulsion | 2016 | To understand how factors associated with person-centred support are experienced in the context of compulsory treatment and to identify good practice in relation to assessment for and management of CTOs. | UK, 1 NHS trust.  Findings based on data from 2 studies.  Participants included service users, relatives and practitioners across teams and the region. | Interviews. | Service users were often inadequately informed about CTO and rights and offered little or no opportunity to make choices or have involvement in CTO process and conditions. Retrospectively they often felt restrictions were beneficial to recovery and reported greater involvement in decisions at review stage. |
| Lawn, S.,  Delaney, T.,  Pulvirenti, M.,  Smith, A.,  McMillan, J. A qualitative study examining the presence and consequences of moral framings in patients’ and mental health workers’ experiences of community treatment orders | 2015 | This paper reports on the moral framing that emerged from the data collected for a broader study of the experience of CTOs from patients’ and workers’ perspectives. | CMHT in Adelaide, South Australia  8 consumers (currently or previously) on CTOs, 10 MH professionals | In-depth interviews. | Experiences of CTOs are multi-layered. Moral framing was used by patients to understand and make sense of the CTO experience and by workers to justify forced care. Empathy and reflection on what is done and how is done is needed. |
| Stuen, HK.,  Rugkasa, J.,  Landheim, A.,  Wynn, R. Increased influence and collaboration: A qualitative study of patients' experiences of community treatment orders within an assertive community treatment setting | 2015 | To report on patients experiences with informal and formal strategies used to promote continued treatment engagement, and to gain insight into how CTOs impact their daily lives. | Norway, 2 urban and 3 rural teams  15 patients seen by an ACT team & on a CTO for at least 6 months. | Qualitative interviews. Methods drew on grounded theory, inspired by constructivist and interpretative framework. | Patients reported mixed responses to CTOs, including a sense of security and feeling violated and controlled. Benefits of support provided by ACT approach were highlighted, including impact of worker and patient relationship on perception of coercion. |
| Light, E.,  Robertson, M.,  Boyce, P.,  Carney, T.,  Rosen, A.,  Cleary, M.,  Hunt, G.,  O'Connor, N.,  Ryan, CJ.,  Kerridge, I. The Many faces of risk: a qualitative study of risk in outpatient involuntary treatment. | 2015 | To derive a model of risk in involuntary outpatient treatment that is meaningful amongst different stakeholders (consumers, carers, MHPs and legal decision makers). | NSW, Australia. 5 patients (currently or previously on a CTO), 6 carers, 12 mental health review tribunal members, 15 clinicians.  Clinicians from outpatient & inpatient across youth, adult, older persons and Aboriginal MH. | In depth semi-structured interviews. Analysis used grounded theory & inductive methods.  Utilised a stakeholder reference group. | There were overlaps among all stakeholders on all discourses on risk in regard to actual harm; social adversity; the system. Clinicians however were influenced by actual harm and questions of risk quantification and carers/consumers with distress of mental illness and related disadvantages. |
| Light, E.,  Kerridge, I.,  Robertson, M.,  Boyce, P.,  Carney, T.,  Rosen, A.,  Cleary, M.,  Hunt, G.,  O'Connor, N.,  Ryan, C. Involuntary psychiatric treatment in the community: general practitioners and the implementation of community treatment orders | 2015 | GPs emerged as a factor in 16 IVs with participants. This paper examines stakeholder perspectives on the GPs role in the area of involuntary treatment.  The larger study examined clinical and legal DM and patient and carer lived experiences aiming to identify potential improvements to CTO processes | NSW, Australia. 2009-2012  5 patients (currently or previously on a CTO), 6 carers, 12 mental health review tribunal members, 15 clinicians  Of the above participant pool: 16 participants (4 clinicians, 2 patients, 6 carers and 4 MHRT member) spoke specifically about GPs | In depth semi-structured interviews. Analysis used grounded theory & inductive methods.  Utilised a stakeholder reference group. | GPs, as primary caregivers, have a significant role in CTOs and value in care provision for people with SMI, though were ‘outsiders’. The lack of integration of GPs in the care of people on CTOs is a shortcoming. |
| Stensrud, B.,  Hoyer, G.,  Granerud, A.,  Landheim, AS. “Life on hold”: a qualitative study of patient experiences with outpatient commitment in two Norwegian counties | 2015 | To examine patients experiences of living with Outpatient Commitment (OC). | 2 counties in Norway.  16 patient participants currently on a OC and with at least 6 months experience of OC. | Grounded theory. Interviews. | The main finding was that of ‘Life on Hold’ reflecting participants’ perceptions that OC prevented them from taking control of their own lives. This was based on perceived coercion; dependence on health care providers & constrained social interaction.  The medical context was perceived as an obstacle to recovery. Some positive experiences identified e.g. feeling safe and secure and easy access to MH staff and services. |
| Stroud, J.,  Banks, L.,  Doughty, K. CTOs: learning from experiences of service users, practitioners and nearest relatives | 2015 | To identify significant issues and good practice in relation to CTOs. | UK, NHS.  72 participants including: service users; care coordinators; responsible clinicians; approved MHPs; service providers & nearest relatives. | Case study. Semi-structured interviews. | Key themes:   1. A CTO provides a legal recognition of need for care 2. CTO provides structure & containment for ‘right’ user 3. Care is defined as predominantly medical 4. There are misunderstanding regarding power and conditions of CTOs   This paper details 1 & 2. |
| Sullivan, WP.,  Carpenter, J.,  Floyd, DF. Walking a tightrope: case management services and outpatient commitment | 2014 | To explore case management practice and case manager perceptions of serving involuntary clients on outpatient commitment orders.  This study was part of a larger qualitative study focused on the role of hopefulness in helping. | USA, Mid-western community mental health service.  19 experienced case managers. | Interviews using an ethnographic method. | Themes included ‘Recipient demand’, ‘OC as a positive tool’, ‘OC as a negative tool’ and ‘minimizing coercive practice’.  The authors suggested advance psychiatric directives and SDM processes can reduce the need for coercive practice. |
| Mfoafa-M’Carthy, M. CTOs and the experiences of ethnic minority individuals diagnosed with SMI in the Canadian MHS | 2014 | Lived experience of individuals from ethnic minority backgrounds who have been subject to CTOs. | Canada, Toronto  24 consumers from an ethnic minority background, currently, or previously on a CTO. | Phenomenology.  Semi-structured interviews. | Positive experiences (affirmation of experiences, improved rapport with case managers & clinical team; increased medication compliance; empowerment) and negative experiences (feeling of coercion and stigma) were reported. |
| Light, E., M.  Robertson, MD.,  Boyce, P.,  Carney, T.,  Rosen, A.,  Cleary, M.,  Hunt, GE.,  O'Connor, N.,  Ryan, C.,  Kerridge, IH. The lived experience of involuntary community treatment: A qualitative study of mental health consumers and carers | 2014 | To describe the lived experience of people subject to CTOs and their carers. | NSW, Australia. 5 patients (currently or previously on a CTO), 6 carers, 12 mental health review tribunal members, 15 clinicians.  Clinicians from outpatient & inpatient across youth, adult, older persons and Aboriginal MH. | In depth semi-structured interviews. Analysis used grounded theory & inductive methods.  Utilised a stakeholder reference group. | Lived experience of CTOs is complex. Reported distress was in part experience of mental illness but also from communication gaps, difficulty getting optimal care and difficulty accessing MHS. It was acknowledged that whilst CTOs are coercive and constrain autonomy they may also be beneficial. This led to an ambivalence about CTOs. |
| Riley, H.,  Hoyer, G.,  Lorem, GF. 'When coercion moves into your home'--a qualitative study of patient experiences with outpatient commitment in Norway | 2014 | To explore patients experiences with Outpatient Commitment (OC) and how routines in care and health services affects patients’ everyday living. | Norway, 11 participants on CTOs for at least 3 months. | In-depth interviews with a narrative approach to interviews and thematic analysis. | Participants generally complied with OC requirements because of a clear and secure framework, but also because of belief that the alternative would be involuntary hospitalisation. Coercion was experienced as a limitation of freedom of action through excessive control and little patient influence of participation in their own treatment. |
| Canvin, K.,  Rugkasa, J.,  Sinclair, J.,  Burns, T. Patient, psychiatrist and family carer experiences of community treatment orders: Qualitative study | 2014 | To examine psychiatrists, patients and family carers experiences of CTOs. | UK.  Part of the OCTET research programme.  75 participants including 26 patients, 25 psychiatrists and 24 family carers.  Participants inclusive of forensic teams. | Grounded theory. In-depth interviews. Data analysed using constant comparative analysis. | All 3 groups perceived main purpose of CTOs as medication enforcement and that the legal clout was central to achieving this.  Understanding of CTO mechanisms varied- uncertainty was expressed about criteria for recall and enforceability of discretionary conditions. There is no single experience or view of CTOs. |
| Ridley, J., Hunter, S. Subjective experiences of compulsory treatment from a qualitative study of early implementation of the Mental Health (Care & Treatment) (Scotland) Act 2003 | 2013 | To explore the experiences and views of individuals who had been treated under the MHCT Act. | Scotland, 49 service users, self-selected who had been under a Care & TO.  Participants included those under inpatient as well as outpatient Care & TOs. 35% clearly community Care & TOs. | Cohort study. Semi-structured interviews.  Interviews were conducted in 2 stages with 80% agreeing to 2^nd^ interview (designed to be 12 months apart). | Legislation had a limited impact on participation in the process of compulsion or change in the dominant psychiatric paradigm. Though service users felt there was increased opportunity for their voices to be heard, this did not result in increased influence over professional decision-making, especially in relation to medication. Fundamental  shifts in practice are needed both in terms of the nature of therapeutic relationships, and in embracing more holistic and recovery perspectives. |
| Gjesfjeld, C., Kennedy, M. Outpatient commitment on the ground: Listening to consumers and providers. | 2011 | To explore the perspectives of consumers and MH providers who are impacted by outpatient commitment. | USA. 9 consumers and 8 service providers (psychiatric nurses, case managers, psychosocial rehabilitation counsellors). | Semi-structured interviews. | Consumers voiced an ambiguous sense of personal control in the context of OPC orders, though reported improvement in their life after being on OPC; consumers and workers had inconsistent understandings of OPC. |
| Schwartz K, O'Brian AM, Morel V, Armstrong M, Fleming C, and Moore P. Community treatment orders: the service user speaks exploring the lived experience of community treatment orders | 2010 | To examine the lived experience of one group of service users on CTOs. | Canada. 6 service users with a SMI and on CTOs. Included consumers seen by 2 ACT teams. | Semi-structured interviews. | There was a lack of focus on conditions & provision of CTO. Issues for participants were less about the CTO and more about labels, control & discrimination associated with SMI. |
| Ridley, J.,  Hunter, S.,  Rosengard, A. Partners in care?: Views and experiences of carers from a cohort study of the early implementation of the Mental Health (Care & Treatment) (Scotland) Act 2003 | 2010 | To explore carers views on the range of compulsory orders.  Part of a larger study to evaluate implementation of the MHCT Act by exploring experiences and perceptions consumers, carers, MH professionals and advocates. | Scotland. 33 carers from three Health Board areas of Scotland as well as the State Hospital.  Carers- predominantly women and parents, 3 were spouses. | Focus groups and individual interviews were conducted with carers at two stages approximately 12 months apart. 8 carers participated at both stages, and 25 carers participated once only. | Many carers felt isolated and unsupported and were critical of the lack of consultation and involvement. Few were aware of carers assessments and many sceptical if this would result in any changes. |
| Gibbs, A. Coping with compulsion: Women’s views of being on a CTO | 2010 | To present the experiences of 10 women either currently or previously on a CTO. To explore benefits & limitations of being on a CTO, support & relationships & d/c experiences. | NZ, Otago. 10 women | Interviews.  Case note review conducted to provide further information about the women. General inductive approach taken for data analysis. | Women experienced benefits and limitations though considered the overall advantages of CTOs to outweigh the disadvantages. Advantages included greater access to treatment and hospital care and an increased sense of safety and reassurance for them & their families. Disadvantages included: some restrictions, such as where they resided; stigma; and having to comply with treatment with the threat of going to hospital if they did not. Overall, CTOs made a significant impact on their lives and allowed them to remain out of hospital, rebuild lives and maintain close relationships. |
| Dawson, J., Mullen, R. Insight and use of community treatment orders | 2008 | To explore the role played by judgements about patients’ insight in reasoning concerning the use of CTOs in NZ. | NZ, Otago. 42 patients with experience or on CTOs, their clinicians (psychiatrists and key workers/case managers) and carers (when possible)  22 patients were on a CTO at the time of IV  90 IVs were conducted with MH professionals (mostly experienced CPNs). 42 with Psychiatrists. | Interviews. Post-hoc analysis of limited data. General inductive approach taken for data analysis. | Lack of insight was an important indicator for compulsory treatment due to perceived link with treatment compliance. Common perception that patients could progressively gain insight during sustained treatment on a CTO. Good insight was not necessarily an indicator for d/c from a CTO if patient posed continuing risks of harm or had a rapid or severe relapse profile.  Potential for treatment compliance appeared to be the primary focus of involuntary treatment decisions. |
| Mullen, R.,  Gibbs, A.,  Dawson, J. Family perspective on community treatment orders: a New Zealand study | 2006 | To explore family members views of use of CTOs. | NZ, Otago. 27 family members. 25 were a spouse or 1^st^ degree relative and 2 were a close friend who had a caring role. | Interviews. General inductive approach taken for data analysis. | Family were generally in favour of CTOs. They perceived positive influences on their relative, themselves, family relationships and relations with the clinical team. Families were aware of ethical and other dilemmas regarding CTO use. |
| Mullen, R.  Dawson, J.  Gibbs, A. Dilemmas for clinicians in use of Community Treatment Orders | 2006 | Clinicians views of the use of CTOs in specific cases in which they were recently or currently involved. | NZ, Otago. Clinicians refer to Psychiatrists (n= 42) who were the ‘responsible clinicians’. | Interviews. General inductive approach taken for data analysis. | Dilemmas were compared with previous literature. The clinicians in the study experienced well known dilemmas such as determining the right for a person’s d/c from CTO, but seemed less troubled by other difficulties than expected as they considered CTOs the best treatment option and best way to manage risks. Further dilemmas identified concerned proper scope of clinical authority over patients on CTOs and decision to revoke CTOs. |
| Gibbs, A.  Dawson, J.  Mullen, R. Community treatment orders for people with serious mental illness: A New Zealand study | 2006 | To examine the views of service users, family members and MHPs about the impact of the CTO regime. | NZ, Otago. 159 participants including service users, their family and treating MHPs. | Semi-structured interviews. General inductive approach taken for data analysis. | Most service users believed the main purpose of CTOs was to ensure medication was taken. They also believed CTOs provided better access to other treatments, supported accommodations and care from MHPs.  Families reported CTOs provided relief and a supportive structure for relatives care.  MHPs found the orders useful for engaging service users in a continuing therapeutic relationship and for promoting treatment adherence. In each group, the majority viewed CTOs as generally positive, whilst acknowledging restrictions it imposed on the persons freedom. |
| O'Reilly, RL.,  Keegan, DL.,  Corring, D.,  Shrikhande, S.,  Natarajan, D. A qualitative analysis of the use of community treatment orders in Saskatchewan | 2006 | To compare views on mandatory outpatient treatment of patients and other stakeholders in Canada with the view of stakeholders from other jurisdictions. | Canada, Saskatchewan. 2 mental health centres.  78 individuals: 14 consumers (12 on CTOs at time of interview), relatives (mostly parents) and MHPs (predominantly nurses). | In-depth interviews and focus groups. | Patients had contradictory feelings about CTOs. Most experienced some degree of coercion while on the orders but many believed that CTOs provided necessary structure to their lives. Clinicians were more consistently positive but recognised the difficult choices in balancing right to self-determination with benefits of CTOs.  Family members viewed CTOs as necessary to control a chaotic situation caused by the persons limited insight. |
| Gibbs, A.,  Dawson, J.,  Ansley, C.,  Müllen, R. How patients in New Zealand view community treatment orders | 2005 | To explore the views of patients with recent experience of CTOs. | NZ, Otago. 42 patients on CTOs, 22 on CTO at time of interview. | Semi-structured interviews. General inductive approach taken for data analysis. | Majority of patients were generally supportive of the CTO, especially if the alternative was hospital. Many valued access to services and sense of security obtained, and attributed improvements in their health to treatment under the order. Experienced reduced choice about medication and restrictions on residence and travel. For a minority this meant they were strongly opposed to CTO, but for most the restrictions did not unduly hinder them. Majority viewed CTO as helpful step towards community stability. |
| Gibbs, A.,  Dawson, J.,  Forsyth, H.,  Mullen, R.,  Tonu Tanga, TO. Maori experience of community treatment orders in Otago, New Zealand | 2004 | To consider the impact of CTOs on Maori patients and their whanau (extended family) and the associated views of MHPs. | NZ, Otago. IVs with 8 Maori patients under compulsory care, their family members and MHPs.  Paper inclusive of 39 IVs. | Semi-structured interviews. General inductive approach taken for data analysis. | Both benefits and drawbacks were identified by patients and family. CTOs were considered helpful in increasing patient safety and whanau security and in promoting access to services. Favoured over hospilitisation, forensic care and homelessness. Drawbacks included sense of external control imposed on both patients and staff, particularly regarding medication and restrictions on choices. |
|  | | | | | |
| **Mixed methods studies** | | | | | |
| **Author and Date** | **Year** | **Aim/Objective** | **Context/Setting** | **Methods and Methodology** | **Findings related to the review** |
| Brophy, L.,  McDermott, F. Using SW theory and values to investigate the implementation of Community Treatment Orders | 2013 | To develop an understanding of good practice from different stakeholder perspectives that inform MH practice with people on CTOs. | Australia, Victoria.  4 consumers, 2 carers, 4 case managers, 4 doctors, MHRB members, senior managers, executive & policy advisors | Different data collection against values of de-individualisation; diversity; equality; empowerment; partnership; social justice & citizenship enabling.  Cluster analysis of 164 people on CTOs; 4 in-depth case studies; semi-structured group interviews. | Importance of carers/family; need for establishment of strong therapeutic relationship; diversity & difference among CTO recipients & purposes or goals of CTO.  5 principles of good practice identified:   1. Use & develop direct practice skills 2. Take a human rights perspective 3. Focus on goals & desired outcomes 4. Aim for quality of service delivery 5. Enhance & enable the role of key stakeholders |
| Taylor, JA.,  Lawton-Smith, S.,  Bullmore, H. Supervised community treatment: Does it facilitate social inclusion? A perspective from approved mental health professionals (AMHPs) | 2013 | To set out the views of AMHPs on the impact of supervised community treatment on their work and their patients’ lives in the community. | UK, 2010  13 AMHPs. | CTO activity, consumer characteristics and conditions of CTO were reviewed.  Questionnaires to 8 AMHPs  Focus group- 5 AMHPs | AMHPs were undecided about benefits of CTOs to their patients. Majority agreed CTOs could benefit patients by earlier identification of relapse, improving access to housing and reducing the risk of harm to self and others. Majority also agreed CTOs had not improved access to employment, education, training or recreational activities, nor helped the stigma and discrimination faced. |
| Brophy, L., Ring, D. The efficacy of involuntary treatment in the community: consumer and service provider perspectives | 2004 | To offer a voice to consumers and service providers about their experiences and views of current practice and policy implementation re CTOs. | Australia, Rural Victoria and Melbourne.  30 consumers and 18 health professionals from a range of professional backgrounds and services participated in interviews/focus groups. | Mostly qualitative approach- focus groups and interviews.  Quantitative data included demographic characteristics of participants and survey responses from health professionals. | Findings suggest that CTOs involve complex decision-making that tests professionals’ ability to make judgements about legal and clinical processes. Consumers were generally dissatisfied with many aspects of the use of CTOs and both groups tended to view CTOs as stigmatising and disempowering. There were a variety of views expressed about the process of admission, discharge, and community supports. |
| Owens, N.,  Brophy, L. Revocation of Community Treatment Orders in a mental health service network | 2013 | An investigation of CTO revocations in a Victorian area MHS. | Australia, Victoria 2008-2010.  Participants included consumer advisory group, local carers and mental health staff. | Data extraction from clinical database; file audit and semi-structured interviews with key-stakeholders. Two different time periods were compared. | CTOs are commonly revoked within 3 months of d/c from inpatient units. Multiple service providers and family/carers have varying involvement that appears to depend on the timing of the referral to the crisis assessment & treatment team.  In the qualitative data there was minimal divergence amongst stakeholder groups. Issues related to care-planning, family involvement and support type were discussed. |
|  | | | | | |
| **Opinion papers** | | | | | |
| **Author and Date** | **Year** | **Aim/Objective** | **Context/Setting** | **Methods and Methodology** | **Findings related to the review** |
| Mfoafo-M'Carthy, M., Shera, W. Beyond community treatment orders: Empowering clients to achieve community integration | 2012 | To review effectiveness of CTOs internationally and specifically in Toronto, Canada. | Canada, Review of literature re history of CTO implementation - brief summaries specific to countries including Australia. | Opinion paper | Advance directives, intensive case management and recovery-orientated service reform are viable empowering alternatives to CTOs. |
| O'Reilly, R.,  Dawson, J.,  Burns, T. Best practices in the use of involuntary outpatient treatment | 2012 | To describe clinicians' views of IOT and reported practices in England, Canada, Australia, and New Zealand. | Draws on research to explore best practice from Commonwealth clinician perspective. | Brief opinion paper | Overall clinicians prefer to mandate only Rx that is known to work and can be delivered with min coercion. Best practice discussed e.g. recall powers, family involvement, service context should be linked, MOUs, resources to provide required services.  D/C indicators discussed & reasons for CTOs. |
| Magnus Mfoafo-M'Carthy, M., Williams. CC. Coercion and community treatment orders (CTOs): One step forward, two steps back? | 2010 | To contribute to a discussion of coercion and its role in  Community MH care, and how it may co-exist with recovery in the implementation of CTOs. | Toronto, international literature discussed in this context. | Opinion paper. | Authors argue that CTOs may not have a place in a recovery-oriented MH care system, though they seem to be a fixed element in current policy. Suggest ways in which they are executed can change to be more recovery-orientated in practice. This includes: consulting with clients about use of the CTO and use to inform recovery plans and advance directives; broaden CTOs to include contractual agreements between clients and workers to include activities to achieve client-determined goals and equal commitment from MHS. |
| Brophy, L., Campbell, J., Healy, B. Dilemmas in the case manager's role: Implementing involuntary treatment in the community | 2003 | To explore the current role of case managers in mental health review board processes, with a particular emphasis on the system in Victoria. | Australia, Victoria. | Opinion paper, augmented with views from case managers from different MHS and different professional backgrounds, carers, policy makers and legal advocates.  Views also sought of attendees at a conference on this theme. | Mental health workers often experience tensions between legal and organisational expectations of their role, their professional orientation and wider understandings of social justice and consumer rights. Case managers in Victoria currently have a limited role within the mental health review board process. Greater involvement of the case manager may enhance the decision-making process of the reviews. |
| Dawson, J.,  Romans, S.,  Gibbs, A.,  Ratter, N.  Ambivalence about community treatment orders | 2003 | Why, despite the gathering momentum of use, does there remain such widespread ambivalence about their use? What are the reasons for this ambivalence? | NZ. | Opinion paper | Summary and critique of literature/empirical evidence. Lack of efficacy and ethical concerns contribute to ambivalence about CTOs. Additional phenomena impacting upon ambivalence include:   - Paradoxes of design - Dilemma of discharge - Volunteers for compulsion |
